# Conservation of Sandy Calcareous Grassland: What Can Be Learned from the Land Use History?

**DOI:** 10.1371/journal.pone.0090998

**Published:** 2014-03-12

**Authors:** Anja Madelen Ödman, Pål Axel Olsson

**Affiliations:** Biodiversity, Department of Biology, Lund University, Lund, Sweden; Roehampton university, United Kingdom

## Abstract

Understanding the land use history has proven crucial for the conservation of biodiversity in the agricultural landscape. In southern Sweden, very small and fragmented areas of the disturbance-dependent habitat xeric sand calcareous grassland support a large number of threatened and rare plants and animals. In order to find out if historical land use could explain variation in present-day habitat quality, the land use on eight such sites was traced back to the 18^th^ century and compared with key factors such as the amount of bare sand, lime content and P availability. There was no support for the common explanation of the decline in xeric sand calcareous grassland being caused by abandonment of agricultural fields during the last century. Instead, fertilization history was the main explanation for the difference in depletion depth of CaCO_3_ seen between the sites. The decline in xeric sand calcareous grassland since the 18^th^ century is most probably the result of the drastic changes in land use during the 19^th^ century, which put an end to the extensive sand drift. Since cultivation was shown to have played an important role in the historical land use of xeric sand calcareous grassland, grazing alone may not be the optimal management option for these grasslands. Instead more drastic measures are needed to restore the high calcium content and maintain proper disturbance levels.

## Introduction

There is a growing awareness among conservation biologists regarding the importance of land use history and local management practices when implementing conservation and restoration measures for biodiversity and threatened species [1], [2], [3]. It is also recognised that land use changes long ago may have caused the vegetation patterns observed today [4], [5]. For example, Eberhardt et al. [2] found that large areas of the sand forests at Cape Cod had been agricultural fields in the middle of the 19^th^ century. They concluded that it was important to “mimic past agricultural practices in order to maintain and restore important sand-plain habitatsÓ. In particular for sandy areas, erosion and vegetation burial have been an important part of habitats history, and plants are adapted to this [6], [7].

Throughout Europe, sandy grassland habitats support a large number of threatened species. Perhaps the best example of such sandy habitats is the threatened xeric sand calcareous grassland (Natura 2000 code 6120, 2002/83/EC Habitat Directive), which is home to several endangered species of vascular plants [8], [9], [10], bryophytes [11], fungi [12] and invertebrates [13]. These habitats are dry, open grasslands on calcareous, more or less humus-free, nutrient-poor and well-drained sandy soils with a discontinuous vegetation cover. The xeric sand calcareous grassland specialists are adapted to high pH [14] and low nutrient content [8], [15], and many of the species depend on the open sand for regeneration [8], [9]. In eastern Skåne (southern Sweden), these grasslands developed on lime-rich glaciofluvial sand [8], [16], which was deposited by glacial meltwater when the ice retreated around 14,000 years ago, and later re-deposited by wind and waves [16]. Sites with natural erosion such as river banks, steep slopes, sand dunes and dune slacks have been proposed as sites where the type of species found in dry grasslands could have occurred before deforestation [17].

**Figure 1:**
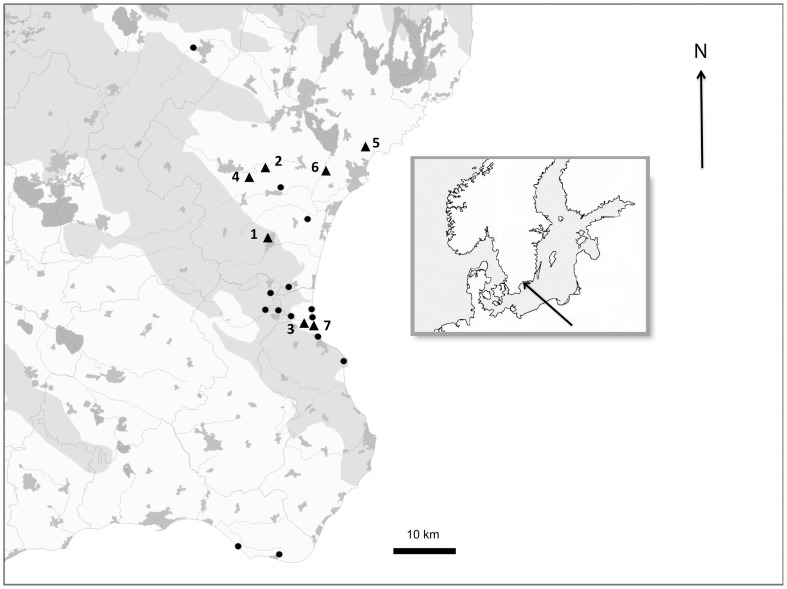
Map showing the existing xeric sand calcareous grassland sites in Skåne. Sites included in the study are represented by triangles and circles represent sites not included. Grey shading indicates forested land and unshaded represents open land. Scale bar represent 10: (1) Degeberga, (2) Everöd, (3) Klammersbäck, (4) Lyngsjö, (5) Rinkaby, (6) Ripa and (7) Vitemölla.

Before 3000 BC, eastern Skåne (Fig. 1) was predominantly covered by forest [18]. After 3000 BC, coppice agriculture led to an opening of the landscape, and there was a concentration of settlements to the flat coastal areas, and around 800 BC large areas of the coastal plains were deforested [18]. From 800 BC, permanent settlements replaced the mobile settlements and fields previously practiced [18]. At this time, intensified land use also opened up the landscape on other places in the Baltic region [19]. An infield/outland system with fertilisation was introduced in the area and there was an expansion of cereal cultivation, which also increased erosion. The traditional method of farming was extensive and with long (up to 30 years) periods of fallow when the arable fields were used for grazing [20], [21], [22]. During the 17^th^ and 18^th^ centuries the human population in southern Sweden grew, which caused further deforestation and intensified agricultural practices [20], [22], and in the 18^th^ century, Linné [23] noted an extensive sand drift and the occurrence of sand fields all over eastern Skåne. The problem with sand drift was for the most part solved at the end of the 18^th^ century and during the 19^th^ century by the introduction of ley plants and more efficient use of fertilisers, which increased the production per surface area and thereby stopped over-exploitation. At the same time, the afforestation of Skåne started [22] and pine was planted to prevent wind erosion. Similar trends with deforestation until the middle of the 19^th^ century, and then afforestation again has also been found in other places in Sweden [24] as well as in New England, USA [25].

Today, the old agricultural practices in sandy grasslands have been abandoned, and the arable fields have been turned into pine plantations or pastures [26], [27], leaving the vegetation cover to close and thereby ending sand drift [28]. A natural accumulation of nutrients and organic matter follows [10], which can be further accelerated by atmospheric nitrogen deposition [29]. In addition, there is a threat of acidification of the topsoil [8]. The remaining areas of xeric sand calcareous grassland are small and fragmented, which poses a further threat to the rarest species (Fig 1).

Linné [23] described a number of xeric sand calcareous grassland species growing in eastern Skåne. In particular *Dianthus arenarius* ssp *arenarius*, which is a key species in these grasslands and protected by the Natura 2000 network (EU Habitat Directive, Annex II), was mentioned several times and was said to grow on all the sand fields, almost like a weed. In fact, all xeric sand calcareous grassland specialists but two, *Silene conica* and *Alyssum alyssoides*, which came to Skåne in the 19^th^ century, were present in Skåne in 1749 [23], [30], [31]. Observations by Linné [23] and Campbell [20] suggest that xeric sand calcareous grassland was widespread in eastern Skåne during the 18^th^ century, but there are no records of the actual area before the 1970s. Since the 1970s, reported areas range between 30 and 50 ha, with no consistent trend between the different studies [26, 27 32]. However, there is a strong indication of decline in xeric sand calcareous grassland species since the 18^th^ century, which is reflected in the high density of nationally red-listed (and thereby declining) plant species [8].

Changes in land use during the last century, i.e. that the agricultural practices on these grasslands were abandoned, has been the common explanation for the decline of xeric sand calcareous grassland in Skåne [26 27, 33]. However, this hypothesis had never been tested prior to this study. The aim of this study was to improve the conservation of dry calcareous grassland by increasing the understanding of how xeric sand calcareous grasslands came to be and why they have disappeared. Land use change and decalcification were compared at eight sites in eastern Skåne in areas with a range of different quantities and qualities of xeric sand calcareous grassland. The amount of bare sand and the CaCO_3_ content of the soil were used as proxies for favourable conditions and, in the case of open sand, as a proxy for erosion. Historical maps were used to determine the former land use and aerial photographs were used to map the extent of bare sand during the last 70 years. By analysing soil profiles, possible links between land use history and decalcification in the studied grasslands were investigated at specific sites within each studied landscape. Extractable P concentrations were also measured as a proxy for fertilisation history, since P remains in the soil for a longer period than N and causes problems during restoration [34], [35].

## Materials and Methods

### Site descriptions

The sites studied were situated in the coastal area of eastern Skåne in southernmost Sweden. The area is 80 km long and 35 km wide, situated between 6172130 and 6204470 N, and 441330 and 458270 E (SWEREF99 TM). Sites were selected on the basis that they contained at least some remnants of xeric sand calcareous grassland vegetation. They should also be, or be part of, a larger open area. Since the focus of the study was the effects of agriculture, sites known to have been created by sand pits or other digging activities were excluded. Still, the majority of landscapes with xeric sand calcareous grassland in the area were included. The sites chosen for the study were: 1. Degeberga (6188081, 442627), 2. Everöd (6198995, 443126), 3. Klammersbäck (61749171, 448414), 4. Lyngsjö (6198615, 442108), 5. Rinkaby (6202616, 455831), 6. Ripa (6198003, 452133) and 7. Vitemölla (6173465, 449873) (Fig 1). The sites are listed in Table 1, where information about their topography, present day management and the amount and quality of the remaining xeric sand calcareous grassland vegetation can be found. These sites are all under nature conservation management, and the conducted study was made in collaboration with the county administrative board in Skåne and followed site-specific regulations. The Everöd, Klammersbäck, Lyngsjö, Rinkaby and Ripa sites are all more or less flat areas. The vegetation at these sites predominantly consists of Fennoscandian lowland species-rich, dry to mesic grassland (N6270, EU Habitat Directive) as well as small areas of heath-like vegetation resembling continental dunes with open Corynephorus and Agrostis grasslands (N2330, EU Habitat Directive) [8], [28]. All of the flat sites contain patches of xeric sand calcareous grassland of varying sizes, depending on the level of degradation. Degeberga and Vitemölla are hilly sites with large presence of xeric sand calcareous grassland vegetation on the south facing slopes, and with Fennoscandian lowland species-rich, dry to mesic grassland on flatter areas.

**Table 1:** A summary of the characteristics of the eight sites analyzed in this study, including the total area analysed and the area of xeric sand calcareous grassland (Natura 2000 habitat 6120) according to Olsson [32].

Site	Topography	Present day management/Disturbance	Area (ha)		Vegetation (vascular plants)				
			Total	Habitat type 6120	Total cover (%)	Specialists cover (%)	Red-listed cover (%)	Red-listed (Nr/m^2^)	N
1. Degeberga	Hilly	Ungulate grazing	13.5	2.6	47±20	23±13	17±14	2.7±0.6	3
2. Everöd	Flat	Ungulate grazing	15	4.9	57±23	14±16	9.2±15	1.0±0.7	10
3. Klammersbäck	Flat	Ungulate grazing	38	0.09	34±16	4±1.4	2.5±3.5	0.5±0.7	2
4. Lyngsjö	Flat	Horse grazing	3	0.1	58±23	12±16	10±16	0.6±0.7	17
5. Rinkaby	Flat	Ungulate grazing/military training	155	4.5	64±20	21±15	14±12	1.4±0.8	25
6a. Ripa airfield	Flat	Fallow/wild grazers (rabbits)	14	0.2	71±23	20±26	17±29	0.3±0.6	3
6b. Ripa pasture	Flat	Ungulate grazing	22	3.0	52±6.4	7.0±5.7	5.5±6.4	1.0±0.0	2
7. Vitemölla	Hilly	Fallow/recreation, wild grazers (rabbits)	38	4.8	59±22	23±21	16±18	1.7±1.3	12

Vegetation data comes from a survey in 2004-2005 [9] and includes the total vegetation cover, as well as the cover of specialist species and nationally red-listed species as defined in Olsson and Ödman [55]. In addition, the number of red-listed species per square meter (Nr/m^2^) is given. Means are displayed (± SD), and N denotes the number of analysed squares (1 m^2^) at each site. The dominant red-listed and specialist species were *Koeleria glauca, Dianthus arenarius* ssp *arenarius* and *Alyssum alyssoides.*

### Soil sampling and analyses

Soil was sampled at the following locations: Everöd (6199099, 443104), Klammersbäck (6174279, 448413), Lyngsjö (6198665, 442138), Rinkaby (6202600, 455808), Ripa pasture (6198191, 452028), and Ripa airfield (6197740, 452277) (SWEREF99 TM). At each of these sites a smaller area was selected that was assumed to consist of degraded xeric sand calcareous grassland. The vegetation at most sampling sites was either Fennoscandian dry to mesic grassland or dry Corynephorus grassland. The soil was sampled on 8-18^th^ June 2009 and soil cores of 20 cm length (∅ 15 cm) were taken down to 1.6 m using a soil auger. Three profiles were sampled at each site, located approximately 10 m from each other in a triangle. At the Ripa airfield site, the soil could only be sampled down to 1.2 m for two of the three profiles because bedrock or boulders were encountered at this depth.

For pH measurements, 10 g of soil was mixed with 50 ml distilled water, and the pH was measured electrometrically in supernatants obtained by 2-h extraction in a rotator. The total amounts of Ca in the samples were measured by digesting the soil in concentrated HNO_3_, followed by ICP-AES analysis. One g of soil at field moisture and 50 ml HNO_3_ were placed in a 300 ml flask, covered with a watch glass and heated on a hot plate for 72 h. The solution was evaporated to 2 ml and diluted to 25 ml with distilled water. Plant-available (extractable) ortho-phosphate (P_ext_) in the top 20 cm was determined using flow injection analysis, with stannous chloride and ammonium molybdate as reactant (measured at 720 nm, method ISO/FDIS 15681-1). Ten g fresh soil was extracted for 1 min with Bray-1 solution [36].

Soil in Degeberga and Vitemölla was not sampled during this study and samples collected for earlier studies by Olsson et al. [8] and Bahr et al. [37] respectively were used instead. At Degeberga, two samples were used: one from Degeberga south slope (xeric sand calcareous grassland) and one from Degeberga north slope (Fennoscandian dry to mesic grassland), which had been sampled down to 1.5 m. At Vitemölla, four samples were used from a mosaic of xeric sand calcareous grassland and dry Corynephorus grassland. The samples were taken down to 0.6 m at three sites and 0.5 m for one of the sites. Data for soil pH and Ca content comes from data collected for earlier studies by Olsson et al. [8] and Bahr et al. [37]. At Vitemölla, Ca concentration values were only available for the top 0.1 m. Additional measurements of P_ext_ were performed on the soil samples for this study, using the methods described above. The samples from the top 0.1 m for all samples at Vitemölla had unfortunately been used up in the previous study, and the 0.1–0.2 m layer was used for the P_ext_ analysis.

### Analysis of land use history

Information on land use in the past was obtained through visual analysis of historical maps and aerial photographs. The area at each site was delimited so that it enclosed the soil sampling plots, had clearly defined borders (arable field margins, roads, water etc.) and only included open land without trees in the 2007 aerial photograph. The sizes of the delimited areas are presented in Table 1. Historical maps were obtained from Lantmäteriet (the Swedish land surveying agency), thru a thorough search in their archives [38]. The historical maps used dated from the middle of the 18^th^ century (oldest maps available from Lantmäteriet) until 1974 and consisted of economic and property redistribution maps (Table 2). The analyses used arable field margins and other objects to locate the sites, and explanatory text or legends were used to determine land use. The land use was either printed on the map or found in a supplementary document accompanying the map. The land uses recorded were: arable field, pasture, meadow, forest and other land uses of interest such as military training or recreation. Areas named “utmark” (outland), “fälad” (old word for common pasture in southern Sweden) and “allmänning” (common land) were classified as pastures, and areas named “wång” (old word for common arable fields in southern Sweden) were classified as arable fields. In the economic map from 1974, only arable fields were mapped and all other land uses were treated as one class (other). The aerial photographs used dated from the 1940s until 2007 (Table 2) and were obtained from Lantmäteriet [39]. The resolution varied between 0.2 m^2^ and 1 m^2^. The analysis, which was done visually, was based on the following criteria: forest (>50% tree cover), arable field (open, with furrows) or pasture (open, without furrows). Furrows were interpreted as signs of ploughing. The map and aerial photograph analyses were performed at each site, both for the whole area (landscape level) and at the exact sites (plot level) where the soil was sampled. The results from the analysis were used to calculate the number of years since the area was last used for cultivation. The number of years was counted from the last year the area was recorded as having been cultivated. In the case of Degeberga and Vitemölla, which had never been cultivated, the number of years from the first available record of land use was used.

**Table 2:** Results from visual analysis of land use history through historical maps (economic and property redistribution maps, with original names in Swedish) and aerial photographs for all eight soil-sampling sites.

	1. Degeberga	2. Everöd	3. Klammersbäck	4. Lyngsjö	5. Rinkaby	6a. Ripa airfield	6b. Ripa pasture	7. Vitemölla
*Aerial photographs*								
**2007**	Pasture	Pasture	Pasture	Pasture	Military training/Pasture	Pasture	Pasture	Pasture/recreation
**2001**					Military training/Pasture			
**1999**	Pasture	Pasture	Pasture	Pasture		Pasture	Pasture	Pasture/recreation
**1985**	Pasture	Pasture	Pasture	Pasture	Military training/Pasture	Airfield/Pasture	Arable field?	Pasture/recreation
**1970**					Military training/Pasture	Airfield/Pasture	Arable field?	
**1969**	Pasture	Pasture	Pasture	Arable field				Pasture/recreation
**1957**	Pasture	Arable field		Arable field		Pasture?	Arable field	
**1956**			Pasture		Arable field			Pasture/recreation
**1940**	Pasture	Arable field	Arable field	Arable field	Arable field	Arable field	Arable field	Pasture/recreation
*Historical maps*								
**1974, Ekonomiska kartan**	Other	Other	Other	Other	Other	Other	Other	Other
**1926-34, Härads ekonomiska**	Pasture	Arable field	Arable field	Arable field	Arable field	Arable field	Arable field	Pasture
**1845, Laga skifte**					Plantation			Meadow, drift sand
**1826, Enskifte**		Arable field		Arable field				
**1822, Enskifte**			Arable field					
**1818, Storskifte**						Arable field	Arable field	
**1811, Enskifte**	Pasture							
**1803, Avmätning**		Arable field		Arable field				
**1751, Ägodelning**					Arable field			Pasture
**Years since cultivation**	196	50	67	38	51	67	22	256

“Other” means an area not classified as arable field or real estate in the economic maps (Ekonomiska kartan). Maps and aerial photographs were obtained from Lantmäteriet [38], [39].

### Analysis of the amount of bare sand and forest

The aerial photographs described above were used to analyse how the amount of bare sand changed between the 1940s and 2007. The aerial photographs were rectified and classified using ArcGIS Desktop 9.3 (ESRI 2008). The delimited areas were the same as those described above for the historical land use analysis. The amount of bare sand was estimated by dividing the surface features shown on the aerial photographs into two classes: bare sand and other (anything which was not bare sand). The classes were identified separately for each photograph using supervised classification where areas of bare sand were identified based on colour (and previous knowledge of the areas for the 2007 photographs), and then used as training areas for the software. The delimited areas were then classified using maximum likelihood classification. The classification for 2007 was evaluated in the field by comparing classified and real patches, and the older ones by comparing the classified patches of bare sand with the visual ones in the aerial photographs. The analysis identified patches of bare sand, but did not consider the overall density of the vegetation. Consequently, only patches with close to 100% bare sand could be identified, and areas with partly bare sand of varying amounts were classified as “other” since they could not be distinguished.

The amount of forest (>50% tree cover) in the surrounding landscape was also analysed from the aerial photographs. The size of the aerial photographs varied considerably between sites and years, and four different areas around the sites, 1×1, 1.5×1.5, 2×2 and 3×3 km, were used. The Everöd and Lyngsjö sites are situated within the same area and were analysed together. Analysis showed that, in those areas where squares larger than 1×1 km could be fitted, the amount of forest for all years increased slightly with increasing size of areas (although not significantly so). However, differences between the years were constant and so only results from the analyses using the 1×1 km squares are presented. Polygons were drawn manually around all forest and the percentage of forest in the total area was calculated using ArcGIS.

### Statistical analyses

Correlations between land use history, amount of forest, amount of bare sand, depletion depth for CaCO_3_, maximum CaCO_3_, pH and P_ext_ content were performed using Pearson correlation or Spearman's correlation when parameters were not normally distributed. For the amount of forest and amount of bare sand, correlations were also performed with year, using the same method as described above. Differences between sites, land use history and depths in terms of the amount of CaCO_3_ and P were tested using a general linear model followed by Tukey's post hoc test. All statistical analyses were performed using IBM SPSS Statistics (IBM Corporation, New York, US). An Arcsine square root transformation of the parameter amount of bare sand was performed to obtain normal distribution. For the correlation between pH and CaCO_3_, the CaCO_3_ values were log transformed. Results presented are mean values and standard deviations.

## Results

### Historical land use changes

The results from the analysis of land use change at the soil sampling plots showed that the last record of cultivation at the six flat sites was between 1940 and 1957, except for the Ripa pasture, where indications of cultivation could be seen also in aerial photographs from 1985 (Table 2). At the sampling plots at the two hilly sites, none of the maps or aerial photographs showed signs of agricultural cultivation (Table 2).

At the landscape level, the six flat areas, Everöd, Lyngsjö, Klammersbäck, Ripa pasture, Ripa airfield and Rinkaby, have all been cultivated but, according to the maps, cultivation ceased between 1940 and the 1990s. They have gone through a historical land use change from a mixture of cultivation and grazing and mowing, to a mixture of forest and grazing. During the 19^th^ century arable fields were common, but at Everöd, Rinkaby and Klammersbäck, pastures occurred as well, and these pastures were managed as commons. At the two coastal sites Rinkaby and Klammersbäck, the common pastures occurred closest to the sea, while fields occurred inland. During the 20th century, the agricultural fields were gradually abandoned and turned into pastures. During this time, forests started to emerge at the Everöd, Rinkaby and Ripa sites.

The two hilly sites, Vitemölla and Degeberga, have a rather similar history. In 1756, the northern part of the site at Vitemölla was classified as very poor arable fields spoiled by sand drift and therefore unusable. The southern part was classified as useless and left as a common in 1778. In 1828 and 1845, the southern part of the area was described as sandy soil and drift sand, and the lower parts close to the sea as useless. According to the maps from 1926-34 and onward, the area was used as pasture and for recreation. The Degeberga site has been used as pasture continually from the first map in 1811 until today. Consequently, according to the maps used in this study, the two hilly sites were not cultivated during the analysed period. However, the note from 1756 about parts of Vitemölla being arable field spoiled by sand drift indicates that some parts had previously been cultivated.

### Forest cover and amount of bare sand from 1940 to 2007 at landscape level

**Figure 2:**
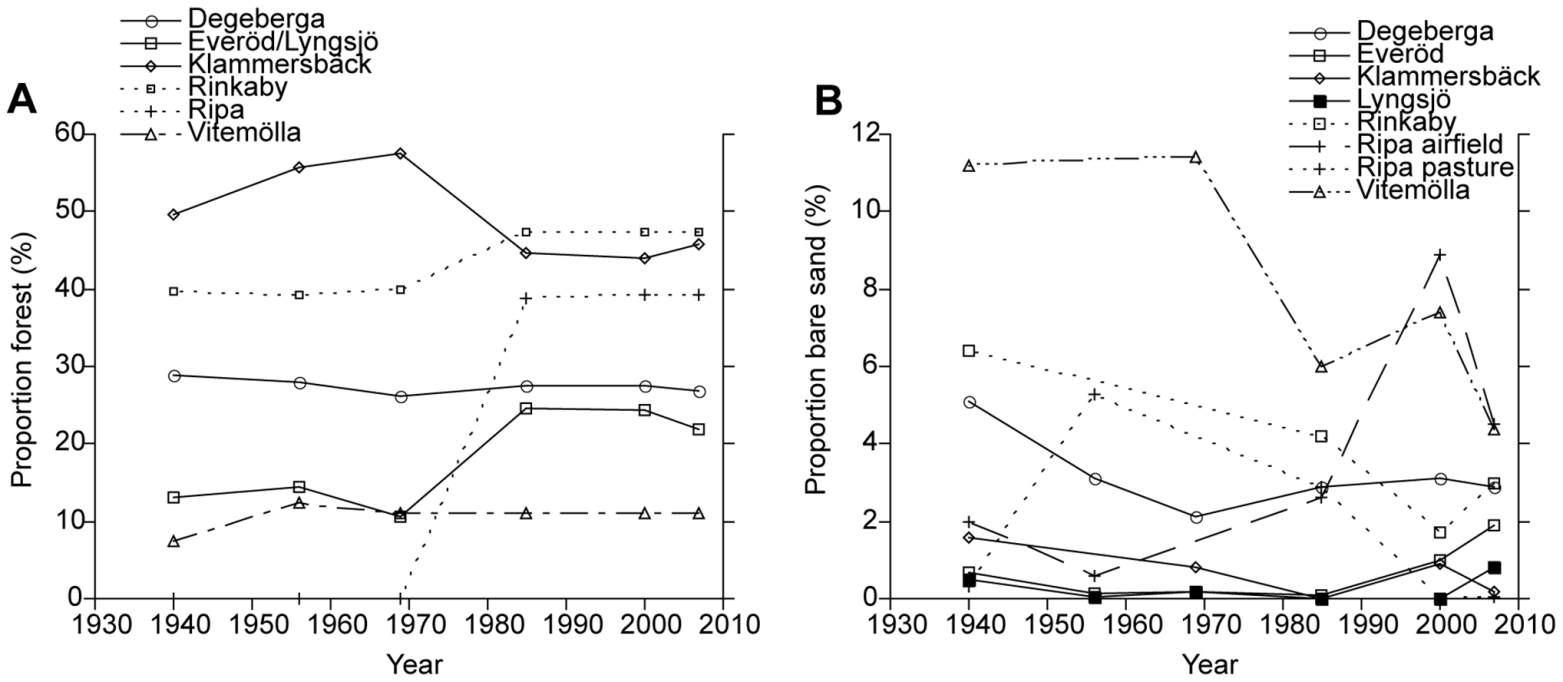
The proportion of land area occupied by forest (a) and bare sand (b) between 1940 and 2009 as analysed from aerial photographs (for simplification we did not differ between 1956/1957 and 1969/1970 in the graph). The proportion of forest was analysed for a 1×1 km square around the sites (Everöd/Lyngsjö and Ripa airfield/Ripa pasture were each contained within the same square), and the proportion of bare sand for the whole area of each of the eight sites.

There was no significant temporal trend in the mean forest cover between 1940 and 2007, when analysed in 1×1 km squares within each landscape. In three areas (Ripa, Everöd/Lyngsjö and Rinkaby) there was an increase in forest cover, while the forest cover decreased in Klammersbäck, and almost no changes could be seen in the other two areas (Fig 2a). The area with the highest mean forest cover over the years (n = 6) was Klammersbäck (50±6%), followed by Rinkaby (44±4%), Degeberga (28±1%), Ripa (20±21%), Everöd/Lyngsjö (18±6%) and Vitemölla (11±2%).

The proportion of bare sand was analysed for eight sites, as Ripa airfield and Ripa pasture were treated separately, ranging in size between 3 and 155 ha. At Vitemölla, Rinkaby and Degeberga, the analysis showed a decrease in the amount of bare sand between 1940 and 2007 (Fig 2b). However, the general trend for all sites was that changes since the 1940s were not drastic, and that the mean amount of bare sand at the studied sites was never more than about 3% of the total study area. There was no significant change in the mean amount of bare sand between 1940 and 2007. The largest change was found at Vitemölla, where the amount of bare sand decreased from 11.2% to 4.4%. There was no correlation between the amount of bare sand and the forest cover or the number of years since cultivation.

### Historical land use and soil properties

**Figure 3:**
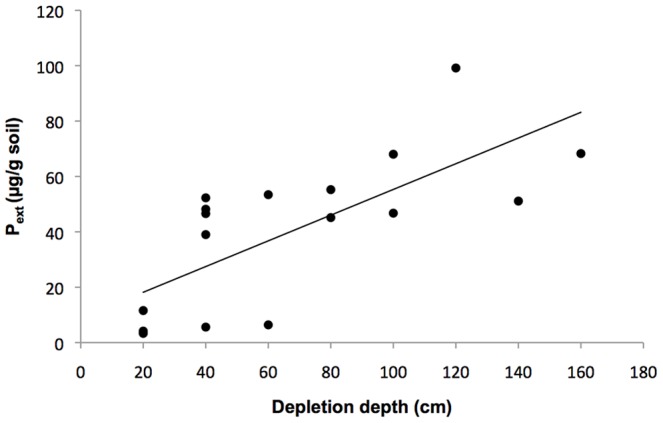
The correlation between P_ext_ (µg g-1 soil) and depletion depth (cm) at the previously cultivated sites (N = 18, R^2^ = 0.53, p<0.001). The number of years since cultivation was counted from the last year the area was recorded as having been cultivated. In the case of Degeberga and Vitemölla the number of years from the first available record of land use was used.

Extractable P (P_ext_) showed large variation between sites. Lyngsjö had the highest mean (n = 3) concentration of P_ext_ (79±18 µg g^−1^ soil), followed by Ripa pasture (51±4.4 µg g^−1^ soil), Ripa airfield (48±4.4 g^−1^ soil) and Everöd (47±6.8 µg g^−1^ soil). Degeberga north (26 µg g^−1^ soil) and the four sampling points at Vitemölla (13±8.2 µg g^−1^ soil) had intermediate concentrations, while the sites at Rinkaby (6.5±4.4 µg g^−1^ soil), Degeberga south (6.3 µg g^−1^ soil) and Klammersbäck (5.1±1.6 µg g^−1^ soil) showed considerably lower P_ext_ values than the other sites. Lyngsjö had significantly higher P_ext_ values than all the other sites (p<0.05) and the Everöd, Ripa pasture and Ripa airfield sites had significantly higher P_ext_ values than Degeberga, Klammersbäck, Rinkaby and Vitemölla (p<0.05). No significant differences were found between the other sites. There was a positive correlation between the P_ext_ and the depletion depth in the sites that had been cultivated (N = 18, R^2^ = 0.53, p<0.001, Fig. 3). There was no significant correlation between the P_ext_ and the number of years since cultivation or the amount of bare sand.

**Figure 4:**
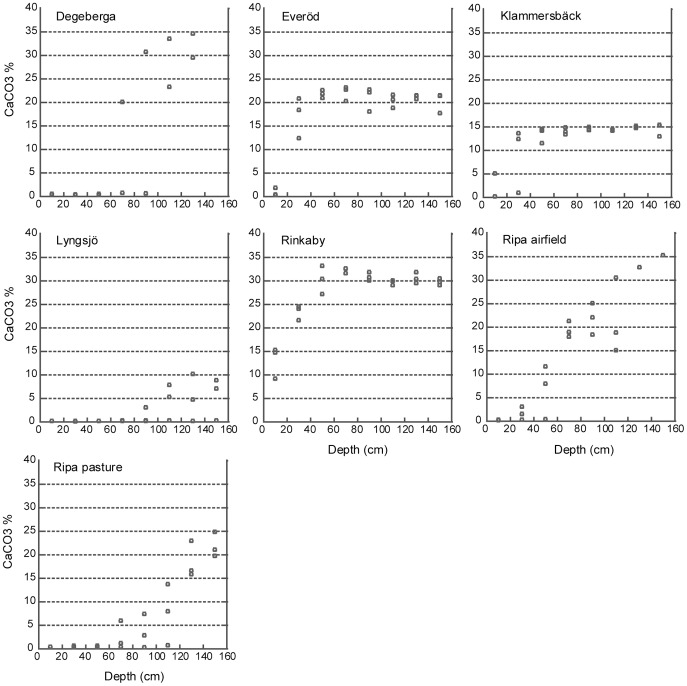
The CaCO_3_ content as percentage of the soil weight for 20 cm at a time down to 160 cm. Vitemölla was not included since samples were only available for the top 20 cm.

None of the sites had an enrichment of CaCO_3_ at any particular depth. Most of the topsoils were depleted but, when the lime horizon was reached, the CaCO_3_ content remained rather constant further down (Fig. 4). The lowest CaCO_3_ values were found at Lyngsjö (0.2±0.03%), which was also the only site where the CaCO_3_ content never exceeded 10%, even in the deepest layer. The only sites with a CaCO_3_ content in the topsoil above 2% were Rinkaby (13.1±3.4%) and Vitemölla (5.4% in sampling point 2), and at 0.2–0.4 m only Everöd, Klammersbäck and Rinkaby had CaCO_3_ concentrations above 2%. The pH values were strongly correlated with the CaCO_3_ values (N = 156, R^2^ = 0.93, p<0.001).

The maximum CaCO_3_ concentration for each profile was identified as well as the depletion depth (the first depth where the CaCO_3_ concentrations exceeded 2%). The maximum CaCO_3_ value was significantly higher at Rinkaby and Degeberga than at Everöd, Klammersbäck, Lyngsjö and Ripa pasture (p<0.05). The maximum CaCO_3_ value at Ripa airfield was significantly higher than that of Lyngsjö (p<0.001), but did not differ from that of Rinkaby and Degeberga. Lyngsjö had a significantly lower maximum CaCO_3_ value than all other sites except Klammersbäck (p<0.001).

There was a positive correlation between the maximum CaCO_3_ and the mean amount of open sand (N = 7, R^2^ = 0.73, p<0.01). The maximum CaCO_3_ value and the depletion depth were not significantly correlated but there was a weak negative correlation between maximum CaCO_3_ and the depth at which the maximum CaCO_3_ was found (N = 20, R^2^ = 0.30, p<0.01). The maximum CaCO_3_ and the pH in the top 20 cm were also positively correlated (N = 20, R^2^ = 0.28, p<0.01). There was no correlation between the number of years since cultivation and the depletion depth, or between the mean amount of bare sand and the depletion depth.

## Discussion

Most of the remaining xeric sand calcareous grasslands in Skåne are found on eroded sites [27], where the underlying lime-rich soil is exposed. This gives an indication about the great importance of erosion and other soil disturbances in the restoration and preservation of these grasslands. The importance of sand pits and military training areas for the conservation of biodiversity and threatened species has also been demonstrated in other parts of Europe [40], [41], [42], [43]. An important finding of this study was that all the flat sites with xeric sand calcareous grassland included in the study had been cultivated in the past, which suggests that former agricultural practices were important in creating the habitat where natural erosion does not occur. Similarly, the chalk grasslands of Salisbury Plain in the UK, which were previously only thought to have a history as sheepwalks, were found to have a history of cultivation [44]. In other types of semi-natural grasslands, grazing continuity is the most important factor for diversity of grazing-dependent species [45], [46]. In the case of sandy grasslands it may instead be that continuous traditional low-intensity cultivation is a very important factor in creating the proper conditions [47], particularly on flat areas where natural erosion is low when covered with vegetation and trampling is not enough to create bare soil. Therefore, the result that disturbance-dependent species were found in areas with long-term continuous soil disturbance created by cultivation should not be surprising, nor should the decline of dry sandy grassland species as soil disturbance decreased.

The cultivation of the soil is thought to have prevented the vegetation cover in the xeric sand calcareous grassland from closing and the CaCO_3_ from being depleted by mixing the soil [26]. The highest average amount of bare sand during the period 1940 to 2007 was just over 3%, and there was no drastic change in the amount of bare sand during this period. The descriptions of Linné [23] and Campbell [20] indicate that there was much more bare sand in the area in the 18^th^ century, and the most important decline probably occurred during the 19^th^ century. Although there was a difference between the sites in amount of bare sand, there was no negative correlation between the amount of bare sand and the number of years since cultivation, which would be expected if lack of cultivation is an important factor in explaining recent declines in xeric sand calcareous grassland species. In fact, one of the sites not cultivated during the last 260 years had the highest amount of bare sand even in the 1940s. It must be acknowledged that there is a problem when comparing flat and hilly sites, as sloping ground will naturally be more prone to soil erosion. However, even when the two hilly sites were removed from the analysis, no correlation between bare sand and number of years since cultivation could be seen. There was a possible constraint to the method used in this study to detect the amount of bare sand. Assuming that cultivation was the important factor and it only occurred every 8–20 years, the analysis made using aerial photographs from just one date every fifteen years in this study could have failed to spot the bare sand produced. Completely bare sand might have been visible only in the first years, although the vegetation cover could have been sparse for many more. This might still have been enough of disturbance to sustain xeric sand calcareous grassland, and the failure to detect semi-bare sand was a weakness in the method used.

Another explanation for the decline in xeric sand calcareous grassland species could be nutrient enrichment. In the present study, some of the more recently cultivated sites had much higher P_ext_ value than sites that were cultivated a long time ago or not at all. However, Rinkaby and Klammersbäck stood out with their low P_ext_ values, suggesting that they have not been fertilized despite their recent cultivation history. The higher P_ext_ values found at some of the more recently cultivated plots could be a reason for the lack of correlation between the amount of bare sand and the number of years since cultivation, since high P values could speed up succession [48], thereby counteracting the effects of soil disturbance. In addition, the fertilisers would also have contained N, which is known to accelerate the decalcification process [49], [50], [51]. This was supported by the positive correlation between P_ext_ and depletion depth of CaCO_3_, which suggests that fertilization could be the main explanation for the difference in depletion depth seen between the sites.

The depletion depth of CaCO_3_ was not correlated with the time since cultivation, which would be expected if the important factor for retention of CaCO_3_ in the topsoil was indeed cultivation. No correlation could be found between the mean amount of bare sand and the depletion depth, indicating a lack of connection between depletion and the amount of soil disturbance during the last century. The positive correlation between P and depletion depth of CaCO_3_ would in fact suggest the opposite, i.e. that cultivation in the last 70 years, if including fertilization, might have had a negative effect. The maximum CaCO_3_ value and the depth at which it was found were negatively correlated, although the correlation was weak. Still, this suggests that the depletion depth of CaCO_3_ partly depended on the soil type. Different glaciofluvial deposits have different CaCO_3_ content depending on the lithology of the local bedrock and the previously deposited soil types [16], which would explain the observed variation in CaCO_3_ content between sites. None of the above-mentioned explanations seems entirely satisfactory in explaining differences in depletion depth between sites and they could not explain the presence of CaCO_3_ in the topsoil of some sites. It is important to keep in mind that depletion has been going on throughout the 14,000 years since the soil was deposited by glacial melt-water. Consequently, there must have been a process counteracting the depletion.

The results discussed above indicate that ploughing was not directly preventing the CaCO_3_ from being depleted. However, it is probable that the wind erosion resulting from the cultivation could have counteracted depletion by exposing the CaCO_3_ rich sand. It seems to be a likely scenario, since wind erosion has already been found to remove fertile topsoil and cause organic matter content to decline [52], [53]. Some sand drift still occurs in southern Sweden [22], but it is limited compared to the extensive sand drift of the 18^th^ century described by Linné [23] and Campbell [20]. The most important factor determining the amount of sand drift is how much bare sand is available for erosion. During the time period from the 1940s until today, the amount of bare sand has been very low, but in the 18^th^ century extensive areas of bare sand were observed due to the intensified cultivation [20], [22], [23]. One other factor that would affect wind erosion is the forest cover in the surroundings. Although the amount of forest at some sites increased during the last 70 years, no correlation was found with the amount of bare sand or the CaCO_3_ content. However, a great change in forest cover took place in the 19^th^ century, when forest was planted to bind the sand [22].

The results strongly suggest that the causes of present-day decline in threatened species may have origins far back in time and that conservation should take this into account. Human activity, such as expansion of arable fields and intensive livestock grazing, also caused sand drift in Germany [9], [10], the UK [54] and the Netherlands [52]. In Germany and the Netherlands, dry sandy grasslands, including xeric sand calcareous grassland and Corynephorus grassland, are believed to have developed in areas where arable farming or livestock grazing created bare sand, which was then exposed to wind erosion and resulted in drifting sand [9], [52]. Today, these sandy grasslands, in common with similar areas in Sweden, are suffering from changes in land use including afforestation, intensification and abandonment [9], [52].

## Conclusions

There was no support for the common explanation of the decline in xeric sand calcareous grassland being caused by abandonment of agricultural fields during the last century, i.e. that the old agricultural practices in these grasslands were abandoned. Instead, fertilization history (as indicated by soil P levels) was the main explanation for the difference in depletion depth of CaCO_3_ seen between the sites. The decline in xeric sand calcareous grassland since the 18^th^ century is most probably the result of the drastic changes in land use during the 19^th^ century, which put an end to the extensive sand drift. The importance of sand movement is shown by the high density of red-listed vascular plant species at the hilly sites (Table 1). Since cultivation played a very important role in the creation of xeric sand calcareous grassland, grazing alone is not a good conservation option for these grasslands [28], [55]. Instead more drastic measures are needed to restore the high calcium content, remove the accumulated nutrients and create bare sand for regeneration [56]. An example of such a restoration measure is topsoil removal, where the decalcified and nutrient rich topsoil is removed much like it would be during sand drift [57]. Not surprisingly, topsoil removal has been shown to be a successful method for restoring xeric sand calcareous grasslands [55].
